# Propan-1-aminium 3,4,5,6-tetra­chloro-2-(meth­oxy­carbon­yl)benzoate

**DOI:** 10.1107/S1600536811004879

**Published:** 2011-02-12

**Authors:** Jian Li

**Affiliations:** a Department of Chemistry and Chemical Engineering, Weifang University, Weifang 261061, People’s Republic of China

## Abstract

In the anion of the title salt, C_3_H_10_N^+^·C_9_H_3_Cl_4_O_4_^−^, the meth­oxy­carbonyl and carboxyl groups are aligned at dihedral angles of 57.8 (3) and 62.5 (3)°, respectively, with the aromatic ring. In the crystal, the cations and anions are linked by N—H⋯O hydrogen bonds, generating chains running aong the *c* axis.

## Related literature

For related structures, see: Li (2011 ▶); Liang (2008 ▶). 
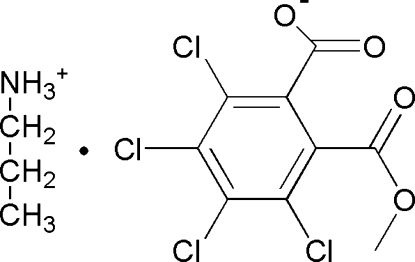


## Experimental

### 

#### Crystal data

C_3_H_10_N^+^·C_9_H_3_Cl_4_O_4_^−^
*M* *_r_* = 377.03
Monoclinic, 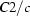

*a* = 28.387 (3) Å
*b* = 14.9600 (13) Å
*c* = 7.8054 (6) Å
β = 93.216 (1)°
*V* = 3309.5 (5) Å^3^
*Z* = 8
Mo *K*α radiation
μ = 0.73 mm^−1^
*T* = 298 K
0.47 × 0.32 × 0.23 mm

#### Data collection

Bruker SMART diffractometer
Absorption correction: multi-scan (*SADABS*; Bruker, 1997 ▶) *T*_min_ = 0.726, *T*_max_ = 0.851
8267 measured reflections
2920 independent reflections
1405 reflections with *I* > 2σ(*I*)
*R*_int_ = 0.069

#### Refinement

*R*[*F* ^2^ > 2σ(*F* ^2^)] = 0.054
*wR*(*F* ^2^) = 0.135
*S* = 1.02
2920 reflections
193 parameters
H-atom parameters constrained
Δρ_max_ = 0.37 e Å^−3^
Δρ_min_ = −0.20 e Å^−3^

### 

Data collection: *SMART* (Bruker, 1997 ▶); cell refinement: *SAINT* (Bruker, 1997 ▶); data reduction: *SAINT*; program(s) used to solve structure: *SHELXS97* (Sheldrick, 2008 ▶); program(s) used to refine structure: *SHELXL97* (Sheldrick, 2008 ▶); molecular graphics: *SHELXTL* (Sheldrick, 2008 ▶) and *PLATON* (Spek, 2009 ▶); software used to prepare material for publication: *SHELXTL*.

## Supplementary Material

Crystal structure: contains datablocks global, I. DOI: 10.1107/S1600536811004879/ng5113sup1.cif

Structure factors: contains datablocks I. DOI: 10.1107/S1600536811004879/ng5113Isup2.hkl

Additional supplementary materials:  crystallographic information; 3D view; checkCIF report

## Figures and Tables

**Table 1:** Hydrogen-bond geometry (Å, °)

*D*—H⋯*A*	*D*—H	H⋯*A*	*D*⋯*A*	*D*—H⋯*A*
N1—H1*A*⋯O4	0.89	2.22	2.938 (4)	137
N1—H1*A*⋯O4^i^	0.89	2.41	2.984 (4)	123
N1—H1*B*⋯O3^ii^	0.89	1.98	2.845 (4)	164
N1—H1*C*⋯O3^iii^	0.89	2.05	2.894 (4)	159

Symmetry codes: (i) 
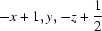
; (ii) 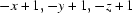; (iii) 
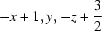
.

**Fig. 1.**
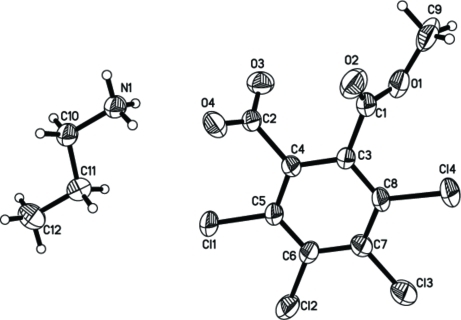
The molecular structure of (I), drawn with 30% probability ellipsoids.

**Fig. 2.**
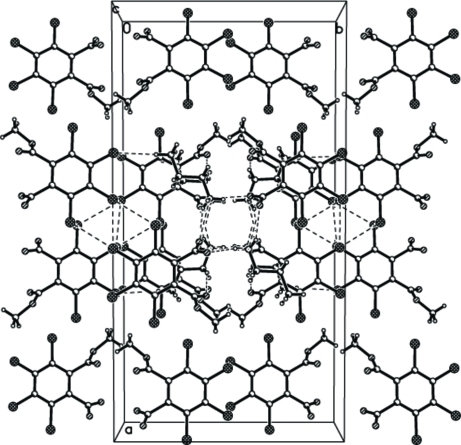
The crystal packing of (I), viewed along c axis. Hydrogen bonds are indicated by dashed lines.

## supplementary crystallographic information

### Comment

We have been studying synthesis of 4,5,6,7-tetrachloro-2-propylisoindoline-1,3-dione. In the present work, the reaction of 2-(methoxycarbonyl)-3,4,5,6-tetrachlorobenzoic acid and propylamine in methanol is expected to yield 4,5,6,7-tetrachloro-2-propylisoindoline-1,3-dione. However, the product is propylaminium 2-(methoxycarbonyl)-3,4,5,6-tetrachlorobenzoate (Scheme I, Fig. 1), the reason may be shorter time and cooler temperature in the reaction. The asymmetric unit of the title compound (I) contains one propylaminium cation and one 2-(methoxycarbonyl)-3,4,5,6-tetrachlorobenzoate anion (Fig. 1). The cation adopts N—C—C—C torsion angle of -178.6 (3) °, the dihedral angles of benzene ring with the methoxycarbonyl and carboxylate groups are 57.8 (3) and 62.5 (3) °, respectively, in the antion. The bond lengths and angles are in agreement with those in ethylammonium 2-(methoxycarbonyl)-3,4,5,6-tetrabromobenzoate methanol solvate (Li, 2011) and in ethane-1,2-diammonium bis(2-(methoxycarbonyl)-3,4,5,6-tetrabromobenzoate) methanol solvate (Liang, 2008). In the crystal structure the cations and anions are connected by intermolecular N—H···O hydrogen bonds into one-dimensional chains along [001](Fig. 2 and Table 1).

### Experimental

A mixture of 4,5,6,7-tetrachloroisobenzofuran-1,3-dione (2.86 g, 0.01 mol) and methanol (15 ml) was refluxed for 0.5 h. And then Propylamine (0.59 g, 0.01 mol) was added to the above solution, being mixed round for 10 min at room temperature. And then the solution was kept at room temperature for 5 d. Natural evaporation gave colourless single crystals of the title compound, suitable for X-ray analysis.

### Refinement

H atoms were initially located from difference maps and then refined in a riding model with C—H = 0.96–0.97 Å, N—H = 0.89 Å, O—H = 0.82Å and *U*_iso_(H) = 1.2*U*_eq_(C) or 1.5*U*_eq_(O, N, methyl C).

### Crystal data

**Table:**

C_3_H_10_N^+^·C_9_H_3_Cl_4_O_4_^−^	*F*(000) = 1536
*M**_r_* = 377.03	*D*_x_ = 1.513 Mg m^−^^3^
Monoclinic, *C*2/*c*	Mo *K*α radiation, λ = 0.71073 Å
*a* = 28.387 (3) Å	Cell parameters from 1429 reflections
*b* = 14.9600 (13) Å	θ = 2.9–23.7°
*c* = 7.8054 (6) Å	µ = 0.73 mm^−^^1^
β = 93.216 (1)°	*T* = 298 K
*V* = 3309.5 (5) Å^3^	Block, colorless
*Z* = 8	0.47 × 0.32 × 0.23 mm

### Data collection

**Table:**

Bruker SMART diffractometer	2920 independent reflections
Radiation source: fine-focus sealed tube	1405 reflections with *I* > 2σ(*I*)
graphite	*R*_int_ = 0.069
φ and ω scans	θ_max_ = 25.0°, θ_min_ = 2.6°
Absorption correction: multi-scan (*SADABS*; Bruker, 1997)	*h* = −23→33
*T*_min_ = 0.726, *T*_max_ = 0.851	*k* = −17→17
8267 measured reflections	*l* = −9→9

### Refinement

**Table:**

Refinement on *F*^2^	Primary atom site location: structure-invariant direct methods
Least-squares matrix: full	Secondary atom site location: difference Fourier map
*R*[*F*^2^ > 2σ(*F*^2^)] = 0.054	Hydrogen site location: inferred from neighbouring sites
*wR*(*F*^2^) = 0.135	H-atom parameters constrained
*S* = 1.02	*w* = 1/[σ^2^(*F*_o_^2^) + (0.0475*P*)^2^] where *P* = (*F*_o_^2^ + 2*F*_c_^2^)/3
2920 reflections	(Δ/σ)_max_ < 0.001
193 parameters	Δρ_max_ = 0.37 e Å^−^^3^
0 restraints	Δρ_min_ = −0.20 e Å^−^^3^

### Special details

**Table:**

Geometry. All e.s.d.'s (except the e.s.d. in the dihedral angle between two l.s. planes) are estimated using the full covariance matrix. The cell e.s.d.'s are taken into account individually in the estimation of e.s.d.'s in distances, angles and torsion angles; correlations between e.s.d.'s in cell parameters are only used when they are defined by crystal symmetry. An approximate (isotropic) treatment of cell e.s.d.'s is used for estimating e.s.d.'s involving l.s. planes.
Refinement. Refinement of *F*^2^ against ALL reflections. The weighted *R*-factor *wR* and goodness of fit *S* are based on *F*^2^, conventional *R*-factors *R* are based on *F*, with *F* set to zero for negative *F*^2^. The threshold expression of *F*^2^ > σ(*F*^2^) is used only for calculating *R*-factors(gt) *etc*. and is not relevant to the choice of reflections for refinement. *R*-factors based on *F*^2^ are statistically about twice as large as those based on *F*, and *R*- factors based on ALL data will be even larger.

### Fractional atomic coordinates and isotropic or equivalent isotropic displacement parameters (Å^2^)

**Table:**

	*x*	*y*	*z*	*U*_iso_*/*U*_eq_	
Cl1	0.50360 (3)	0.81553 (7)	0.52072 (18)	0.0958 (5)	
Cl2	0.55595 (5)	0.99424 (7)	0.6113 (3)	0.1375 (7)	
Cl3	0.66359 (4)	0.99436 (7)	0.6998 (2)	0.1342 (7)	
Cl4	0.72101 (4)	0.81799 (7)	0.68593 (18)	0.0961 (5)	
N1	0.44237 (10)	0.58365 (18)	0.4533 (4)	0.0630 (9)	
H1A	0.4637	0.6137	0.3968	0.095*	
H1B	0.4443	0.5257	0.4286	0.095*	
H1C	0.4480	0.5915	0.5657	0.095*	
O1	0.68601 (9)	0.63305 (17)	0.7732 (4)	0.0749 (8)	
O2	0.66423 (10)	0.5995 (2)	0.5002 (4)	0.0908 (10)	
O3	0.56435 (9)	0.59345 (17)	0.6786 (4)	0.0749 (8)	
O4	0.54157 (9)	0.63029 (17)	0.4104 (4)	0.0710 (8)	
C1	0.66366 (13)	0.6473 (3)	0.6217 (7)	0.0604 (11)	
C2	0.56159 (12)	0.6450 (2)	0.5521 (6)	0.0530 (10)	
C3	0.63685 (11)	0.7345 (2)	0.6219 (5)	0.0527 (10)	
C4	0.58827 (12)	0.7341 (2)	0.5810 (5)	0.0522 (9)	
C5	0.56380 (12)	0.8150 (2)	0.5743 (5)	0.0665 (12)	
C6	0.58697 (14)	0.8952 (2)	0.6135 (6)	0.0797 (14)	
C7	0.63492 (14)	0.8952 (2)	0.6518 (6)	0.0779 (13)	
C8	0.66020 (12)	0.8156 (2)	0.6525 (5)	0.0619 (11)	
C9	0.71825 (15)	0.5576 (3)	0.7804 (7)	0.1017 (16)	
H9A	0.7007	0.5031	0.7637	0.153*	
H9B	0.7352	0.5562	0.8904	0.153*	
H9C	0.7402	0.5635	0.6919	0.153*	
C10	0.39403 (12)	0.6172 (2)	0.4012 (5)	0.0634 (11)	
H10A	0.3714	0.5914	0.4756	0.076*	
H10B	0.3856	0.5984	0.2846	0.076*	
C11	0.39174 (14)	0.7179 (2)	0.4118 (5)	0.0731 (12)	
H11A	0.4139	0.7436	0.3354	0.088*	
H11B	0.4010	0.7367	0.5278	0.088*	
C12	0.34187 (16)	0.7531 (3)	0.3628 (6)	0.0944 (15)	
H12A	0.3335	0.7382	0.2454	0.142*	
H12B	0.3413	0.8168	0.3767	0.142*	
H12C	0.3197	0.7262	0.4357	0.142*	

### Atomic displacement parameters (Å^2^)

**Table:**

	*U*^11^	*U*^22^	*U*^33^	*U*^12^	*U*^13^	*U*^23^
Cl1	0.0429 (6)	0.0713 (7)	0.1698 (14)	0.0085 (5)	−0.0226 (7)	−0.0073 (7)
Cl2	0.0788 (9)	0.0531 (7)	0.276 (2)	0.0161 (6)	−0.0340 (11)	−0.0109 (9)
Cl3	0.0782 (9)	0.0619 (7)	0.259 (2)	−0.0211 (6)	−0.0221 (10)	−0.0134 (9)
Cl4	0.0379 (6)	0.0906 (8)	0.1587 (13)	−0.0099 (5)	−0.0041 (7)	−0.0034 (8)
N1	0.053 (2)	0.0559 (18)	0.080 (2)	−0.0011 (15)	0.0030 (17)	−0.0029 (17)
O1	0.0562 (17)	0.0818 (19)	0.085 (2)	0.0207 (14)	−0.0071 (16)	0.0062 (16)
O2	0.087 (2)	0.090 (2)	0.094 (3)	0.0328 (17)	−0.0111 (18)	−0.0228 (19)
O3	0.085 (2)	0.0574 (17)	0.083 (2)	−0.0072 (14)	0.0046 (16)	0.0042 (16)
O4	0.0572 (17)	0.0811 (19)	0.073 (2)	−0.0115 (14)	−0.0083 (15)	−0.0113 (16)
C1	0.041 (2)	0.059 (3)	0.081 (4)	0.0008 (19)	−0.001 (2)	0.002 (2)
C2	0.035 (2)	0.050 (2)	0.075 (3)	−0.0007 (17)	0.005 (2)	0.000 (2)
C3	0.039 (2)	0.048 (2)	0.071 (3)	0.0034 (18)	−0.0019 (18)	0.0013 (19)
C4	0.042 (2)	0.048 (2)	0.067 (3)	−0.0035 (18)	−0.0029 (18)	0.0037 (19)
C5	0.038 (2)	0.054 (2)	0.106 (4)	0.0011 (18)	−0.008 (2)	−0.001 (2)
C6	0.047 (3)	0.049 (2)	0.141 (4)	0.0082 (19)	−0.010 (3)	−0.002 (2)
C7	0.053 (3)	0.049 (2)	0.130 (4)	−0.009 (2)	−0.009 (3)	0.002 (2)
C8	0.033 (2)	0.062 (2)	0.090 (3)	−0.0047 (18)	−0.003 (2)	0.003 (2)
C9	0.071 (3)	0.101 (3)	0.132 (4)	0.040 (3)	−0.002 (3)	0.032 (3)
C10	0.050 (2)	0.059 (2)	0.081 (3)	−0.0034 (18)	0.000 (2)	−0.002 (2)
C11	0.076 (3)	0.062 (3)	0.081 (3)	−0.005 (2)	0.000 (2)	0.002 (2)
C12	0.080 (3)	0.087 (3)	0.115 (4)	0.022 (3)	−0.005 (3)	0.005 (3)

### Geometric parameters (Å, °)

**Table:**

Cl1—C5	1.736 (3)	C4—C5	1.396 (4)
Cl2—C6	1.724 (4)	C5—C6	1.393 (5)
Cl3—C7	1.723 (4)	C6—C7	1.377 (5)
Cl4—C8	1.732 (3)	C7—C8	1.391 (5)
N1—C10	1.496 (4)	C9—H9A	0.9600
N1—H1A	0.8900	C9—H9B	0.9600
N1—H1B	0.8900	C9—H9C	0.9600
N1—H1C	0.8900	C10—C11	1.510 (5)
O1—C1	1.327 (5)	C10—H10A	0.9700
O1—C9	1.453 (4)	C10—H10B	0.9700
O2—C1	1.189 (4)	C11—C12	1.538 (5)
O3—C2	1.251 (4)	C11—H11A	0.9700
O4—C2	1.234 (4)	C11—H11B	0.9700
C1—C3	1.511 (5)	C12—H12A	0.9600
C2—C4	1.544 (5)	C12—H12B	0.9600
C3—C8	1.396 (4)	C12—H12C	0.9600
C3—C4	1.398 (4)		
			
C10—N1—H1A	109.5	C7—C8—C3	120.2 (3)
C10—N1—H1B	109.5	C7—C8—Cl4	119.5 (3)
H1A—N1—H1B	109.5	C3—C8—Cl4	120.2 (3)
C10—N1—H1C	109.5	O1—C9—H9A	109.5
H1A—N1—H1C	109.5	O1—C9—H9B	109.5
H1B—N1—H1C	109.5	H9A—C9—H9B	109.5
C1—O1—C9	115.3 (3)	O1—C9—H9C	109.5
O2—C1—O1	126.0 (4)	H9A—C9—H9C	109.5
O2—C1—C3	123.4 (4)	H9B—C9—H9C	109.5
O1—C1—C3	110.7 (4)	N1—C10—C11	111.2 (3)
O4—C2—O3	127.1 (3)	N1—C10—H10A	109.4
O4—C2—C4	118.8 (4)	C11—C10—H10A	109.4
O3—C2—C4	114.1 (4)	N1—C10—H10B	109.4
C8—C3—C4	119.7 (3)	C11—C10—H10B	109.4
C8—C3—C1	121.1 (3)	H10A—C10—H10B	108.0
C4—C3—C1	119.1 (3)	C10—C11—C12	111.7 (3)
C5—C4—C3	119.1 (3)	C10—C11—H11A	109.3
C5—C4—C2	120.3 (3)	C12—C11—H11A	109.3
C3—C4—C2	120.5 (3)	C10—C11—H11B	109.3
C6—C5—C4	120.7 (3)	C12—C11—H11B	109.3
C6—C5—Cl1	119.7 (3)	H11A—C11—H11B	107.9
C4—C5—Cl1	119.6 (3)	C11—C12—H12A	109.5
C7—C6—C5	119.8 (3)	C11—C12—H12B	109.5
C7—C6—Cl2	120.0 (3)	H12A—C12—H12B	109.5
C5—C6—Cl2	120.2 (3)	C11—C12—H12C	109.5
C6—C7—C8	120.2 (3)	H12A—C12—H12C	109.5
C6—C7—Cl3	119.8 (3)	H12B—C12—H12C	109.5
C8—C7—Cl3	120.0 (3)		
			
C9—O1—C1—O2	7.9 (6)	C4—C5—C6—C7	2.9 (7)
C9—O1—C1—C3	−171.6 (3)	Cl1—C5—C6—C7	−178.3 (4)
O2—C1—C3—C8	−119.1 (4)	C4—C5—C6—Cl2	−178.0 (3)
O1—C1—C3—C8	60.5 (5)	Cl1—C5—C6—Cl2	0.8 (6)
O2—C1—C3—C4	57.3 (6)	C5—C6—C7—C8	−0.4 (7)
O1—C1—C3—C4	−123.1 (4)	Cl2—C6—C7—C8	−179.5 (4)
C8—C3—C4—C5	−1.0 (6)	C5—C6—C7—Cl3	179.9 (4)
C1—C3—C4—C5	−177.4 (4)	Cl2—C6—C7—Cl3	0.7 (6)
C8—C3—C4—C2	−178.1 (4)	C6—C7—C8—C3	−2.8 (7)
C1—C3—C4—C2	5.5 (6)	Cl3—C7—C8—C3	176.9 (3)
O4—C2—C4—C5	64.5 (5)	C6—C7—C8—Cl4	175.4 (4)
O3—C2—C4—C5	−117.7 (4)	Cl3—C7—C8—Cl4	−4.9 (6)
O4—C2—C4—C3	−118.5 (4)	C4—C3—C8—C7	3.5 (6)
O3—C2—C4—C3	59.3 (5)	C1—C3—C8—C7	179.8 (4)
C3—C4—C5—C6	−2.2 (6)	C4—C3—C8—Cl4	−174.7 (3)
C2—C4—C5—C6	174.9 (4)	C1—C3—C8—Cl4	1.6 (5)
C3—C4—C5—Cl1	179.1 (3)	N1—C10—C11—C12	−178.6 (3)
C2—C4—C5—Cl1	−3.8 (5)		

### Hydrogen-bond geometry (Å, °)

**Table:**

*D*—H···*A*	*D*—H	H···*A*	*D*···*A*	*D*—H···*A*
N1—H1A···O4	0.89	2.22	2.938 (4)	137
N1—H1A···O4^i^	0.89	2.41	2.984 (4)	123
N1—H1B···O3^ii^	0.89	1.98	2.845 (4)	164
N1—H1C···O3^iii^	0.89	2.05	2.894 (4)	159

Symmetry codes: (i) −*x*+1, *y*, −*z*+1/2; (ii) −*x*+1, −*y*+1, −*z*+1; (iii) −*x*+1, *y*, −*z*+3/2.
